# Community perspectives on treating asymptomatic infections for malaria elimination in The Gambia

**DOI:** 10.1186/s12936-019-2672-7

**Published:** 2019-02-18

**Authors:** Fatou Jaiteh, Yoriko Masunaga, Joseph Okebe, Umberto D’Alessandro, Julie Balen, John Bradley, Charlotte Gryseels, Joan Muela Ribera, Koen Peeters Grietens

**Affiliations:** 1 Medical Anthropology Unit, Institute of Tropical Medicine, Antwerp, Belgium; 2 Medical Research Council Unit the Gambia at the London, School of Hygiene and Tropical Medicine, Fajara, The Gambia; 3 Faculty of Social and Behavioural Science, Department of Sociology and Anthropology, University of Amsterdam, Amsterdam, The Netherlands; 4 PASS Suisse, Neuchâtel, Switzerland; 5 London School of Hygiene and Tropical Medicine, London, UK; 6 School of Health and Related Research (ScHARR), The University of Sheffield, Sheffield, UK

**Keywords:** Asymptomatic infection, Malaria, Elimination, Community perspectives

## Abstract

**Background:**

Innovative and cost-effective strategies that clear asymptomatic malaria infections are required to reach malaria elimination goals, but remain a challenge. This mixed methods study explored people’s attitudes towards the reactive treatment of compound contacts of malaria cases with a 3-day course of dihydroartemisinin-piperaquine (DHAP), the socio-cultural representations of asymptomatic infections, and more specifically their treatment.

**Methods:**

Prior to the start of the intervention, a sequential mixed method study was carried out. Qualitative data collection involved in-depth interviews and participant observations (including informal conversations) with key informants from the trial communities and the trial staff. Quantitative data were derived from a pre-trial cross-sectional survey on health literacy and health-seeking behaviour among randomly selected members of the study communities.

**Results:**

In the pre-trial cross-sectional survey, 73% of respondents reported that malaria could be hidden in the body without symptoms. Whilst this may be interpreted as people’s comprehension of asymptomatic malaria, qualitative data indicated that informants had different interpretations of asymptomatic disease than the biomedical model. It was described as: (i) a minor illness that does not prevent people carrying out daily activities; (ii) an illness that oscillates between symptomatic and asymptomatic phases; and, (iii) a condition where disease agents are present in the body but remain hidden, without signs and symptoms, until something triggers their manifestation. Furthermore, this form of hidden malaria was reported to be most present in those living in the same compound with a malaria case (71%).

**Conclusion:**

Treating asymptomatic malaria with pharmaceuticals was considered acceptable. However, people felt uncertain to take treatment without screening for malaria first, largely due to the lack of symptoms. Knowledge of asymptomatic malaria was not a strong re-inforcement for treatment adherence. In this study, the pre-intervention active engagement of communities existed of having people co-design accurate information messages about their personal risk of malaria, which increased their trust in expert knowledge and thus proved essential for the successful implementation of the community-based intervention.

## Background

As global strides are made towards malaria elimination, the focus of interventions has shifted to include a group of people identified as ‘asymptomatic carriers’ and/or as the ‘human reservoir of infection’ [1]. The latter are defined as malaria-infected but apparently healthy individuals who may be the source of infection for malaria vectors, thereby maintaining transmission [1–4]. In clinical malaria studies, ‘apparently healthy’ usually means the absence of fever, the most apparent symptom of malaria [5, 6]. Although for other vector-borne diseases, such as dengue, the term ‘inapparent symptomatic’ is often used [7–9] to implicate the possibility of asymptomatic carriers to encounter (mild) symptoms, the clinical spectrum of *experienced* asymptomatic malaria has not yet been clearly defined or elaborated on. In contrast to symptomatic infections, asymptomatic malaria infections indeed tend to generally be described microbiologically. As such, an asymptomatic malaria case exhibits a considerably lower parasite density than a symptomatic case crossing the threshold of what is detectable by currently available diagnostic methods such as light microscopy or a rapid diagnostic test (RDT) [10, 11]. Research shows that low density infections, including sub-microscopic infections, could be important contributors to malaria transmission in areas with very low transmission intensity [10, 12, 13]. In the context of malaria elimination goals, the possibility of continuous transmission maintained by undetected malaria cases highlights the significance of understanding the ‘human reservoir of infection’ [10, 11, 14].

At low malaria prevalence, identifying asymptomatic carriers becomes increasingly difficult because of the need of screening many individuals to identify a few infected ones. Ultra-sensitive molecular methods can maximize the detection of lower densities of parasites, but are currently not feasible in routine surveillance [11]. A possible approach would be to treat the whole population, regardless of malaria infection status, with an efficacious anti-malarial, i.e. mass drug administration (MDA). However, with the currently available treatments, this approach automatically excludes some population groups, such as pregnant women or infants under 6 months old, while other groups, such as mobile populations, would be easily missed. Furthermore, both MDA and alternatives such as scheduled screening and treatment (SST) are difficult to sustain over long periods of time, particularly when transmission has decreased to very low levels, due to population fatigue, logistical challenges and costs. Targeting sub-groups or geographical areas may be an alternative approach but has similar shortcomings [1, 5, 6]. Lastly, reactive case detection (RCD), a strategy in which household members of a passively identified clinical malaria case are screened and treated if positive, has the limitation of the current diagnostic tools in detecting low density infections [15]. Furthermore, RCD may not be sustainable in the short or long term in low to moderate transmission settings [16] as it may require active commitment of the populations involved [17].

To overcome these limitations, reactive household-based, self-administered treatment (RHOST) was tested through a cluster-randomized trial in a low prevalence setting of The Gambia. The concept and the trial protocol have been published elsewhere [18]. The trial proposed an innovative approach for targeting the human reservoir of infection by combining: i) the passive detection of clinical malaria cases; ii) systematic treatment without screening with dihydroartemisinin-piperaquine (DHAP) of all individuals living/sleeping in the index case’s compound; and, iii) an active community participation by means of involving patients, their households and other community actors in tackling implementation concerns and co-designing solutions according to their knowledge and experience.

One of the key challenges for the optimal impact of interventions targeting asymptomatic individuals is their adherence to medication while not being apparently sick [19]. As a malaria elimination programme in the Solomon Islands showed, despite community engagement and education, people were unwilling to take drugs on repeated MDA rounds because they were not sick [20]. As other studies have also shown [21–26], understanding and consequently adapting intervention strategies to people’s perceptions of asymptomatic infections is crucial for the effectiveness of such community-based interventions. Complex biomedical concepts such as asymptomatic infections are difficult to elaborate on with the communities targeted for MDA when using only biomedical language [22, 23, 27, 28].

Perceptions of malaria in sub-Saharan Africa have frequently been shown to be different from the biomedical model, consequently influencing the uptake of interventions [29–32]. Furthermore, concepts from traditional and biomedical knowledge sources can syncretize to produce hybridized perceptions of malaria [33, 34]. These findings have stimulated the development and inspired the design of culturally appropriate information, education and communication (IEC) strategies to improve intervention uptake. However, it is also widely acknowledged that improving knowledge through well designed IEC strategies does not automatically lead to changed health behaviour [34]. There is limited understanding on how asymptomatic malaria is understood by recipients of MDA, and how these perceptions affect the implementation and/or the uptake of the intervention. This study, set within the context of a cluster-randomized trial that included key community participation and engagement component, focused on understanding and contextualizing the perceptions of asymptomatic malaria and its treatment.

## Methods

### Study setting

#### Study population

This mixed methods study was carried out in the North Bank Region of The Gambia. The main ethnic groups residing in these villages are Fula, Mandinka and Wollof, although there are other minority groups: Bambara, Tilibonka and Turkas, that migrated from neighbouring Mali and Burkina Faso. Most are Muslims. Each ethnic group speaks their own language, although Mandinka or Fula is the common language for communication and trade. Farming is the most common occupation, with peanuts as the main cash crop, while rice, maize, beans, and vegetables are grown for own consumption. A significant number of families supplement their income through remittances from relatives living either in the major towns or abroad.

#### Malaria context

Malaria transmission in the area is seasonal, low (transmission prevalence of 5%) and occurs mostly between July and December. The main malaria parasite is *Plasmodium falciparum* [35].

#### Trial context

The trial was a cluster-randomized trial designed to assess the impact of reactive treatment of contacts of a clinical malaria case living in the same compound on the prevalence of malaria infection at the end of the transmission season. It was planned over two transmission seasons. In the first season (preparatory phase), study villages (17 intervention, 17 control) were identified and approaches to integrate the intervention into the communities and health system were tested and adapted by social scientists and health system researchers through formative research. During this process, trial implementation concerns were identified and solutions co-developed with relevant stakeholders, including community members, health service providers and policy makers. The final health messages for sensitization, including the medicine distribution strategy involving key community actors such as village health workers (VHWs) and compound heads developed for community members to support the intervention, were applied in all intervention villages in the second season (implementation phase).

### Study design

As part of the formative research during the preparatory phase, a sequential exploratory mixed-methods study was conducted, in standard annotation (QUAL → quan) [36]. In the first qualitative strand (March–May 2016), ethnographic techniques were used in all intervention villages (n = 17) to understand the general socio-cultural, political and economic context, including an overview of all health care options in the region. People’s perceptions on asymptomatic malaria were also explored during this phase. Based on the qualitative findings, a questionnaire was developed for the cross-sectional survey in the sequential quantitative strand (June 2016). The survey assessed the representativeness of the qualitative findings in all the study villages (n = 34).

### Qualitative strand

#### Participants and sampling

In the qualitative study, sampling was theoretical meaning that informants were purposefully and gradually selected based on emerging findings. Access to the informants was usually granted through snowball sampling, where initial participants are used to identify and connect the researchers with other theoretically relevant informants. The *Alkalo* (village head) was a starting point to gradually and purposefully select other people from the study villages, including recent malaria cases (individuals diagnosed by rapid diagnostic test (RDT) by a health worker) and their caretakers, compound heads, VHWs and *marabouts* (traditional healers). From the trial team, study nurses and fieldworkers were also included. Sampling aimed for maximum variation to ensure that all relevant groups in relation to the research question were included.

### Data collection

#### In-depth interviews

Data collection began by carrying out in-depth interviews with the *Alkalos* who were identified as gatekeepers to the communities and knowledgeable on the general socio-cultural, political, economic, and pluralistic medical context. In their roles as gatekeepers and source of reference, they identified households recently affected with malaria and/or other key informants, which helped to build trust with the interviewees. The focused inquiry with key informants involved the in-depth exploration of topics such as the concept of asymptomatic malaria infection, the processes and metaphors used to describe asymptomatic conditions, and the acceptability of treating asymptomatic individuals with anti-malarials. Interviews were conducted by the authors (FJ and JM) with the help of two translators. The interviews were recorded and transcribed verbatim by the translators. Additionally, during interviews, field notes were written by FJ and JM for immediate analysis.

#### Participant observation

During the fieldwork, informal conversations were conducted with community members, trial nurses and fieldworkers. These conversations were useful to confirm or refute emerging findings and generate new hypotheses for testing.

### Data analysis

Data analysis was a flexible and iterative process. Preliminary data from in-depth interviews and participant observations were jointly analysed by FJ and JM to further adapt the interview guide and process. Additional data collection was used to confirm or refute temporary findings until the point of saturation (i.e. no new findings emerging). All interviews were systematized and analysed with NVivo 11 Qualitative analysis software (QSR International Pty Ltd, Cardigan UK).

### Quantitative strand

#### Participants and sampling

Participants were identified using a bootstrap sampling technique with the population stratified by household and age (children (< 16 years) and adults). The sampling frame ensured that the number of households selected was in proportion to the size of the village. The questionnaire for children was administered to caregivers and where any of the initially selected persons were unavailable, the next person in the household in the same age category was approached to complete the questionnaire.

### Data collection

A paper-based questionnaire containing closed-ended and open-ended questions was used to collect quantitative data. Topics included health-seeking behaviour for malaria, health literacy including knowledge on asymptomatic malaria carriers, adherence to malaria treatment and trust in different health providers. The survey was piloted by the social science team to ensure clarity and to avoid translation errors in the three main languages. Survey data were collected by the social science team and trial fieldworkers previously trained on interview techniques and administering questionnaires with closed and open-ended questions.

### Data analysis

All completed survey forms were double entered in Excel by trial data entry clerks, verified by the social science team and cleaned using Epi Info. Data were analysed using STATA 15 (Stata Corp, Tx). For this manuscript, frequencies for variables related to asymptomatic carriers are presented. These are presented stratified by gender, ethnicity, education level and socio-economic status. Chi squared tests were used to assess whether differences were statistically significant. Socio-economic status was calculated from questions related to education, housing, income and employment using principal component analysis.

### Ethical approval

Ethical approval was obtained from the joint Gambia Government/Medical Research Council Unit The Gambia Ethics Committee and the Institutional Review Board of the Institute of Tropical Medicine, Antwerp, Belgium. The interviewers followed the Code of Ethics of the American Anthropological Association. All interviewees were informed before the interview about the topic and types of questions and their right to decline participation, to interrupt or withdraw from the conversation. Oral consent was sought before each interview and was documented by the interviewer. Oral consent was favoured since the risk was minimal and the act of signing one’s name on a document was expected to cause mistrust towards the study team since this is not customary practice within the local communities. Interviewees confidentiality was assured by assigning unique identifiers to the collected forms.

## Results

### Study participants

#### Qualitative strand

In-depth interviews (n = 88) and informal conversations (n = 5) were carried out with community members and field staff (Table 1).

**Table 1:** Overview of respondents for in-depth interviews and informal conversations

Participants	In-depth interviews	Informal conversations
Village heads (*Alkalos*)	8	
*Alkalo’s* brother	2	
*Alkalo’s* son	1	
*Alkalo’s* wife	1	
*Marabout* (healer)	2	
Malaria case caregiver (women)	2	
Farmer (men)	42	
Farmer (women)	30	
Field staff (men)		5
Total	88	5

#### Quantitative strand

In total, 741 questionnaires were completed; there were no refusals; 324 adults and 417 children (< 16 years) questionnaires were completed. The questionnaire for children (< 16 years) was administered to their caretakers (Table 2).

**Table 2:** Socio-demographic characteristics respondents in quantitative study (N = 741)

	n	%
Gender		
Male	308	41.6
Female	423	57.1
Missing	10	1.3
Ethnicity		
Mandinka	385	52.0
Fula	221	29.8
Wolof	50	6.7
Tilibonka	34	4.6
Bambara	27	3.6
Turkai	5	0.7
Other	19	2.6
Knows how to read (English)		
Yes	108	14.5
No	630	85.0
Occupation (adults and caretakers of participating minors [< 16])		
Farmer	673	90.8
Public officer	6	0.8
Business	41	5.53
Other	21	2.83

#### Conceptualization of asymptomatic carriage

There were no local terms in the commonly spoken local languages, i.e. *Mandinka, Fula* and *Wolof*, corresponding directly to ‘asymptomatic disease’. Consequently, asymptomatic disease was explained as a health condition where a disease is present, but the affected person does not feel any signs and symptoms related to the disease. Based on this explanation, respondents interpreted the statement to mean a health condition where disease was present in the body but did not affect one’s appetite nor restricted their ability to carry out daily activities. The condition, to which everyone was considered susceptible, was understood as covering the following scenarios: (i) a minor illness which mild symptoms does not prevent people from carrying out daily activities; (ii) an illness that oscillates between symptomatic and asymptomatic phases; and, (iii) a condition where disease agents are present in the body but remain hidden until something triggers their manifestation in apparent symptoms. During the asymptomatic phase, the so-called ‘body soldiers’ (i.e. a representation of antibodies provided in health messages) are fighting the hidden disease but produce no apparent symptoms. Symptoms only appear when the body soldiers are defeated in the presence of external triggering factors. The elaborated local model of illness progression for asymptomatic conditions therefore presents the following characteristics: (i) everyone is susceptible to an asymptomatic condition; (ii) the disease can remain hidden for a variable period, depending on the fight in the body (body soldiers); (iii) symptoms will manifest when body soldiers are defeated or when certain triggers occur; (iv) disease can be transmitted even in asymptomatic form; and, (v) asymptomatic conditions should be treated when they become symptomatic.

#### Perceptions of asymptomatic malaria

Several diseases (biomedical) and folk illnesses (illnesses that do not correspond to any biomedical classifications of diseases), such as yellow fever, worms, lung tuberculosis, *jinn* (spirits) afflictions and malaria, were considered having asymptomatic stages within the local explanatory model of health and illness. However, these distinct biomedical conditions were not always perceived to be distinct locally and were reported to naturally evolve into one another.

Malaria was described as one of the conditions that could be asymptomatic. The majority of respondents (73%) thought that malaria can be hidden in the body without showing symptoms (Table 3). The proportions of those who thought so were similar across gender, ethnicity, educational level and socio-economic status. Respondents in the survey considered this form of hidden malaria most likely to be present in those living in the same household with a malaria case (71%), with no statistically significant differences across the socio-demographic characteristics (Table 3). Similarly, 78% of respondents stated that living in the same household as someone having malaria increased their personal risk of getting malaria. This varied by whether the respondent thought malaria could be asymptomatic, with 80% of those who thought malaria could be asymptomatic considering they were at increased risk as compared to 69% of those who did not (p = 0.002). 91% of respondents reported having a mosquito net in the home, and this did not vary by whether the respondent thought malaria could be asymptomatic or not (91%) and 88% of net use respectively (p = 0.168).

**Table 3:** Perceptions of asymptomatic infections in relation to socio-demographic characteristics [% of respondents affirming the statement (n/N)]

	Hidden malaria in the body	Increased risk in same household	Mosquito bites trigger symptoms	Hot sun triggers symptoms	Drinking milk triggers symptoms
Total	73% (538/736)	71% (416/586)	89% (520/585)	91% (533/587)	85% (498/586)
Gender					
Male	74% (225/306)	71% (172/243)	87% (210/242)	89% (216/243)	85% (206/243)
Female	73% (306/421)	71% (240/336)	91% (306/336)	92% (310/337)	85% (286/336)
p-value	0.800	0.865	0.100	0.205	0.909
Ethnicity					
Mandinka	74% (285/383)	74% (218/296)	90% (267/296)	95% (283/297)	87% (257/297)
Fula	73% (159/219)	67% (125/186)	88% (163/185)	83% (155/186)	82% (152/185)
Wolof	60% (30/50)	58% (19/33)	79% (26/33)	91% (30/33)	82% (27/33)
Other	76% (64/85)	76% (54/71)	90% (64/71)	92% (65/71)	87% (62/71)
p-value	0.162	0.108	0.245	< 0.001	0.517
Education level					
None	72% (380/530)	70% (290/416)	88% (366/415)	91% (378/417)	85% (355/417)
Primary	78% (113/144)	76% (91/119)	92% (109/119)	90% (107/119)	84% (100/119)
Secondary	76% (29/38)	63% (20/32)	88% (28/32)	100% (32/32)	87% (27/31)
p-value	0.242	0.207	0.563	0.182	0.905
Socio-economic status quartile					
Wealthiest	80% (143/178)	73% (108/148)	92% (139/151)	89% (135/152)	84% (128/152)
2nd	73% (132/180)	73% (105/144)	88% (117/132)	91% (121/133)	81% (107/132)
3rd	69% (124/179)	68% (91/133)	88% (128/145)	97% (140/145)	89% (129/145)
Poorest	73% (133/181)	69% (105/152)	87% (129/148)	88% (130/148)	86% (127/148)
p-value	0.118	0.745	0.562	0.043	0.313

Qualitative interviews indicated that asymptomatic malaria was perceived to be hidden in different parts of the body and frequently in the head and joints where pain is first experienced.

*“Generally, diseases can be housed in different parts of the body. Most times, for malaria the head is the problem and it starts with that place.”* (Adult man, Farmer)

The time it takes for malaria symptoms to appear varied depending on a combination of factors such as: (i) one’s ability to withstand or cope with illness represented by the concept of ‘body strength’ and, (ii) the duration the body soldiers of the affected individual fights. For instance, the period of pregnancy was considered a phase of low body strength and thus pregnant women were perceived to have weaker body soldiers. Following these logics, the strength of the body (i.e. blood or body soldiers) can be increased or nourished by consuming certain foods such as green leafy vegetables and decreased through hard work and taking cold baths. This illustration reinforces the notion that one is asymptomatic when the body is strong and can thus hide the disease. When the body is weakened by the culmination of a set of external triggers (i.e. sun, milk, hard work), the symptoms of the disease can no longer be hidden and start to emerge. Most of the respondents (91%) thought that if malaria was hidden in the body, hot sun can make the symptoms appear. This perception significantly varied across ethnicity (Mandinka (95%), Wolof (91%) and Fula (83%), *P *< 0.001), and socio-economic levels (wealthiest quartile (89%), 2nd quartile (91%), 3rd quartile (97%) and poorest quartile (88%), p = 0.043) (Table 3). Among those who thought that if malaria was hidden in the body, 85% reported that drinking milk could make the symptoms appear, with similar proportions across socio-demographic characteristics.

It was widely acknowledged that mosquitoes could directly cause malaria (99.8%) and increased malaria risk was associated with mosquito density, which was related to stagnant water. Another commonly mentioned way of contracting malaria was ‘body heat’ transfer, understood as the transmission of heat (and disease) through direct (e.g. sleeping together with a malaria case) or indirect (e.g. sleeping in the place where a malaria case person was sleeping before) contact. In addition, the dormant state of malaria could be triggered to an active state by a mosquito bite (89%) (with similar proportions across the socio-demographic characteristics), resulting in apparent symptoms such as fever, headache, vomiting and body aches.

When malaria is related to and often even equated with the folk illness *joontinoji* (Fula), it is perceived to progress from its symptomatic phase to yellow fever (*Chawnabee*-Fula).

*“When malaria has power, it can change into yellow fever”* (Adult farmer, Talen Fula).

This progression was said to be more common during the rainy season. Yellow fever, locally referred to as *Chawnabee* (Fula), *Sumuyaa* (Tilibonka), *Sai* (Tilibonka) and *Paices* (Wollof), was believed to affect mostly children. The symptoms (yellowing of the eyes and vomit, fever, the changing colours of the palms of the hands) appear temporarily and then the disease hides again. The appearance of the symptoms is triggered by the consumption of specific food items, such as milk, which were more commonly available during the rainy season. During the symptomatic phase the disease can be transmitted to others by looking into the sick person’s eyes.

### Perceptions of anti-malarial treatment for asymptomatic carriers

#### Diagnosis and uncertainty

Although most people accepted that malaria could be asymptomatic, people do not actively seek treatment when symptoms are absent. People perceived analgesics (paracetamol or ibuprofen) taken after experiencing mild malaria symptoms to strengthen the body enough so it can hide the remaining malaria. Such hidden malaria is then considered undetectable by biomedical diagnostic tools, such as RDTs.

#### Anti-malarial treatment for asymptomatic malaria

Informants generally considered it unnecessary to take medication without having any symptoms. However, when anti-malarial treatment is given after screening and the individual found to be positive, it was perceived to be a necessary means of disease prevention. This is consistent with local practices of self-protection with amulets or charms commonly referred to as *fankanta* (Mandinka) to prevent ailments such as *jinn* afflictions, common childhood illnesses or the ‘evil eye’ during women’s pregnancy. Once anti-malarial medication is consumed after screening, the medication is perceived to increase the body’s strength, enabling it to engage in a fight with the disease. In contrast to analgesics, malaria medication strengthens the body to the point where it can eliminate the disease rather than hide it. During this process, the blood becomes hot, making people sweat. Observed sweating after taking anti-malarial treatment was therefore seen as evidence of treatment efficacy.

*“If you take anti*-*malarial treatment, it goes into the blood to fight what it is meant to fight. If you take the pills, it goes through the blood, which gets hot to fight the disease and when you sweat, then they say that you are feeling better from malaria and the medicine is good for you.”* (Adult man, Farmer)

The efficacy of the anti-malarial medication was also perceived to be contingent on the prior ‘body strength’ of the individual. As a result, there was a general notion that individuals differed substantially and therefore that the treatment might work for some (eliminating the disease) and fail for others (hiding the disease only). The failure of treatment for some individuals was also attributed to the perceived strength of the anti-malarial medication in relation to its dosage. Especially taking an incomplete anti-malarial regimen could trigger the symptoms to reappear.

### Attitudes towards health workers

77% of respondents said they received good or very good advice from the VHWs. This varied by whether the respondent thought malaria could be asymptomatic, with 73% of those who thought malaria could be asymptomatic receiving good or very good advice, compared to 86% of those who did not (p = 0.002). 95% of respondents said they received good or very good advice from the personnel at the health facility. This did not vary by whether the respondent thought malaria could be asymptomatic, with 95% of those who thought malaria could be asymptomatic receiving good or very good advice, compared to 93% of those who did not (p = 0.235). Qualitative data further showed that trust in health workers (VHWs and health facility personnel) was relatively high. However, the main limitation for VHWs which generated distrust in the community was the common complaint that the provision of RDTs and treatment was continuously deteriorating.

## Discussion

This study explored how local perspectives on asymptomatic carriage were structured in relation to specific health interventions. Perceptions of asymptomatic carriage reflect a dynamic understanding of health and illness, where every individual is vulnerable to hidden conditions where disease agents are constantly perceived to be engaged in a battle with the body’s defences (i.e. body soldiers). If the body soldiers are defeated, the body’s ability to resist or cope with illness (body strength) diminishes, leading to manifestation of the symptoms of the disease. Similarly, in a study carried out on perceptions of vaccinations in The Gambia, medical interventions such as immunizations were perceived as protecting the health of the child by giving ‘strength’ or ‘power’ to withstand or cope with illness [37].

For some conditions such as yellow fever and malaria, the manifested symptoms are seen as interchangeable, thereby representing the transformative nature of asymptomatic conditions. The perceived elements making up the disease process inside the body, mirrors both components from local knowledge systems related to ‘hidden diseases’ described elsewhere [25, 29, 37, 38], and biomedical health information related to the body’s defence mechanism with antibodies. Indeed, in the study area, there has been a long presence of health campaign groups, including malaria programmes, that use metaphoric terms such as body soldiers in their health promotion activities in order to explain the process of natural immunity against parasites and other invading pathogens in the body [37]. Furthermore, the knowledge on body soldiers are conceptually linked to more general notions of body and blood strength. Following these logics, the act of consuming certain foods such as green leafy vegetables to strengthen the body soldiers to withstand illness could be compared to the perceived protective nature of pharmaceuticals and vaccinations to strengthen the body.

In relation to asymptomatic malaria and its treatment, this merging of different knowledge systems is neither arbitrary nor incoherent, as it follows an internal logic of disease transformations (from asymptomatic to symptomatic, malaria to yellow fever), which was key for understanding people’s acceptance of the condition as relevant. These findings also support recommendations to move away from the term ‘asymptomatic’ malaria in favour of ‘chronic malaria’ [10], or even ‘inapparent symptomatic’ as used for other infections [9]. Such a reconceptualization would allow for a clinical spectrum of mild symptoms, disease progressions and associated co-morbidities, which the assumption of a ‘benign’ asymptomatic infection without any clinical symptoms does not currently support.

### Implications for MDA strategies including reactive mass treatment

The perception of malaria to be potentially asymptomatic was relatively high with most respondents believing that living in the same household as someone having malaria increased their personal risk of getting malaria. Qualitative data supported illness progression pathways where malaria is perceived to hide, grow and eventually manifest when triggered by external factors. This concept of illness progression was often used to explain events *post facto* or for narrative purposes and was therefore not a strong determinant for adhering to malaria preventive measures. The asymptomatic infection was a key cause of diagnostic uncertainty, which has been described to occur in situations where the decision-maker cannot assign definite values to objects and events and/or an individual is unable to predict outcomes because sufficient cues are lacking [39]. Because they had a clear understanding of malaria symptoms, the lack of these symptoms in asymptomatic phases led to a state of uncertainty exemplified by respondents’ insistent questions of how they could be aware of having the disease when they did not experience any symptoms. Previous studies on asymptomatic conditions have shown that uncertain outcomes related to one not knowing whether they had a disease that was asymptomatic were reasons for treatment refusal or abandonment [25, 40].

However, unlike the findings of the malaria elimination programme in the Solomon Islands where asymptomatic individuals were not considered as sick and refused treatment, most of our informants ascertained anti-malarials to cure malaria even if the disease was in a dormant phase. Furthermore, the study population showed willingness to accept RDT positive results in the absence of symptoms. Since 2008, there has been a significant roll-out in The Gambia of RDTs with artemisinin-based combined therapy (ACT) including the availability and accessibility to long-lasting insecticide-treated bed nets (LLINs) and indoor residual spraying (IRS) for malaria control [41]. These developments are reported to have contributed to the reduction of malaria burden [35]. Although current conventional tests cannot detect asymptomatic infections, the positive perceptions held by this population on the accuracy of positive diagnostic results highlight the need for the continuous strengthening of health worker capacity, especially VHWs to effectively manage malaria cases.

In the situation of diagnostic uncertainties highlighted by this study, the authority of and trust in an expert may become more important than people’s perceptions of health and illness. This warrants an active engagement of communities in research projects in order to strengthen their trust in biomedical specialists’ diagnosis and health information on particular malaria risks in this context. This further relates to the expectation that when pursuing malaria elimination, programmes will find themselves working in and with communities that progressively adapt their perceptions of malaria risk to decreasing incidence [42]. Interventions designed to actively engage communities could provide an opportunity to re-establish or heighten awareness of malaria risk and thereby foster trust in health authorities.

### Strength and limitations

This study is the first in-depth analysis of community’s understanding on the concept of asymptomatic malaria infections and its implication for malaria elimination in West-Africa. There is evidence from South East Asia and East Africa depicting populations misunderstandings on asymptomatic malaria infections and willingness to take treatment in MDAs [25, 26, 43]. A recent publication from Laos in the context of antimalarial resistance in the Greater Mekong sub-Region highlights the need for MDA interventions with accompanying community engagements to build trust with different stakeholders [26]. By using a mixed-methods approach our work demonstrated the logic behind the study population’s understanding of asymptomatic infections in order to highlight before implementation, the facilitators and barriers of adherence for improving acceptability of the strategy. The study population’s acknowledgement of the increased risk of asymptomatic malaria for individuals sharing the same compound with a symptomatic malaria case (71%) could be influenced by pre-intervention information spread through the community, nonetheless, it signifies the relevance of understanding perceived malaria transmission dynamics.

## Conclusion

This study on perspectives of asymptomatic malaria infections in The Gambia highlights that people’s perception of illness progression could make elimination strategies targeting asymptomatic individuals acceptable in the local context. However, due to the uncertainty of future manifestations of malaria symptoms, these perceptions do not currently act as a motivation for treatment adherence. To address the issue of diagnostic uncertainty, elimination strategies will need to actively engage communities to foster trust in expert knowledge.
